# Beyond natural targets: chemical synthesis reprograms the target specificity of rapamycin

**DOI:** 10.1093/nsr/nwac039

**Published:** 2022-03-03

**Authors:** Ziyang Zhang

**Affiliations:** Department of Cellular and Molecular Pharmacology, University of California, San Francisco, USA

Natural products have served as a rich source of new drugs, either directly or as inspirations for pharmacophores [1]. Under the constant pressure of evolution, natural products have exploited numerous biosynthetic machineries to achieve their extraordinary chemical diversity as well as unique mechanisms of action. The macrocyclic immunosuppressant rapamycin provides a case in point [2]. As a potent (sub-nanomolar) inhibitor of the protein kinase mTOR, rapamycin exerts its pharmacological function with an unusual mechanism—it binds to the abundant cellular protein FKBP12, and the resulting binary complex engages mTOR to form an inhibitory ternary complex. In doing so, rapamycin acts as a molecule glue, forging an unnatural protein–protein interaction as its therapeutic mechanism. Similar mechanisms are also utilized by other natural product immunosuppressants such as FK506, cyclosporin and sanglifehrin.

**Figure 1.**
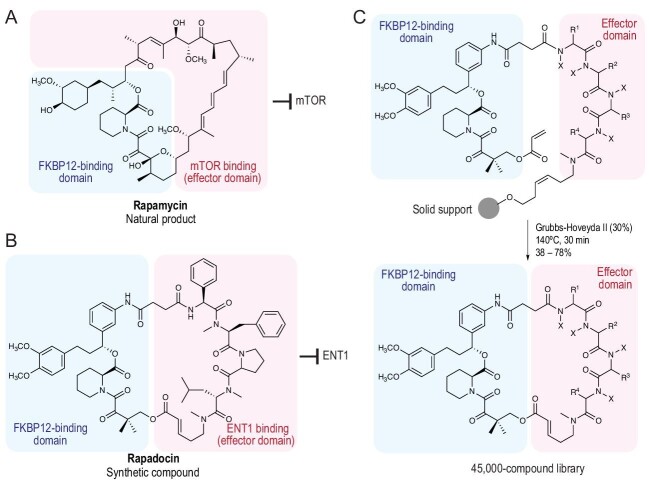
(A) Structure of rapamycin. (B) Structure of rapadocin. (C) Construction of FKBP-binding macrocycles with ring-closing metathesis with concomitant release from solid support. In each structure, the FKBP12-binding domain and effector domain are indicated with blue and red shades, respectively.

Structurally, rapamycin features distinct motifs that contribute to its binding to FKBP12 (FKBP-binding domain) and its interaction with mTOR (effector domain) (Fig. 1A). This molecular anatomy led to the hypothesis that one might reprogram the therapeutic mechanism of rapamycin to engage other biological targets by chemically modifying the effector domain of rapamycin. However, discovery of such compounds with neomorphic functions represents a formidable challenge for at least two reasons: (i) the efficient synthesis of analogs of rapamycin, which possesses a macrocyclic scaffold and a daunting array of stereocenters, requires innovative strategies and precise chemical manipulations; (ii) protein–protein interactions are sensitive to minute structural perturbations, and finding compounds that induce unnatural interactions likely requires sampling a large chemical space.

Jun Liu and co-workers sought to tackle this challenge by building a rapamycin-like macromolecule library with diverse effector domains [3]. The authors developed a general method to rapidly assemble FKBP-binding macrocycles by solid-phase synthesis (Fig. 1C). A library of random tetrapeptide was synthesized as the effector domain using the split-pool strategy, where an olefin-containing linker was placed between the solid-phase support and the peptide. Each peptide was then joined with a constant FKBP-binding domain (FKBD) by amide coupling. The inclusion of a terminal olefin on the FKBD allowed a ring-closing metathesis reaction to take place in the presence of Hoveyda–Grubbs catalyst II, furnishing a 34- or 36-membered macrolactone with its concomitant release from the solid-phase support. Both the FKBD and the linker were heavily optimized for optimal FKBP12-binding and cyclization efficiency, respectively.

The authors constructed a 45 000-compound library and used it in a cell-based screen for inhibitors of nucleoside transport. Three out of these 45 000 compounds in the same structural family exhibited sub-micromolar potency; chemical optimization of these compounds led to the discovery of ‘rapadocin’ (Fig. 1B), which had nanomolar cellular potency (IC_50_ = 5.0 nM) without detectable toxicity. Rapadocin inhibited nucleoside transport by forming a ternary complex between FKBP12 and the equilibrative nucleoside transporter ENT1. Interestingly, the authors also observed FKBP12-independent binding between rapadocin and ENT1, although the interaction was much stronger with the participation of FKBP12.

ENT1 is a crucial protein in adenosine signaling and a validated therapeutic target of drugs known as adenosine reupdate inhibitors. The authors demonstrated, in mouse models, that by inhibiting ENT1 and thus sustaining extracellular adenosine levels, rapadocin was able to protect against ischemic reperfusion injury, a common surgery complication.

Modification of natural products by chemical synthesis represents a powerful method of accessing uncharted chemical space. Such modifications often impart improved properties (e.g. potency, selectivity) to the parent molecule and sometimes overcome resistance mechanisms [4]. Yet rarely are these modifications found to create neomorphs, i.e. to completely change the biological target. The study by Liu and co-workers offers a compelling demonstration of how chemical synthesis can complement biosynthesis and expand the target landscape of natural products. As recent work using other chemical platforms [5] and genome mining approaches [6] reveal the potential of neomorphic rapamycin analogs, it is reasonable to believe that members of this 45 000-compound library could emerge as exciting probes or drug candidates for other important disease targets.
